# Probiotics and prebiotics: health claim substantiation

**DOI:** 10.3402/mehd.v23i0.18568

**Published:** 2012-06-18

**Authors:** Seppo Salminen, Henk van Loveren

**Affiliations:** 1 Functional Foods Forum, University of Turku, Turku, Finland; 2 Laboratory for Health Protection Research, The National Institute of Public Health and the Environment, Bilthoven, The Netherlands; 3 Department of Toxicogenomics, Maastricht University, Maastricht, The Netherlands

**Keywords:** claims, regulation, health benefits, probiotics

## Abstract

‘Probiotics’ and ‘prebiotics’ by definition should have health benefits. Health claims on microorganisms proposed as probiotics and probiotic stimulating agents (prebiotics) suggest that there is a relationship between the specific food and maintaining good health or that the food can reduce the risk of a disease. The Health Claim Regulation in European Union aims at a level consumer protection. Thereby, health claim assessment focuses on defining the probiotics and prebiotics, assessing the health relationship and evaluating studies with emphasis on controlled human intervention studies. The challenges include the focus of claims for healthy populations while most intervention studies with probiotics and prebiotics have been conducted in patients or subjects at risk of specific diseases. Another challenge is the risk reduction claim, which requires demonstrated changes in biomarkers that are generally accepted as indicators of disease risk. Existing assessment opinions from EFSA illustrate the need for further research for probiotics and prebiotics in the future.

The Regulation on Health and Nutrition Claims aims to ensure that consumers are not misled by false, ambiguous, or misleading claims. With the current legislation, consumers should be able to rely on clear and accurate information on food labels, enabling them to be properly informed on the food they choose. The background information and the categories of health claims and the review of scientific data on existing claims in different EU member states (Article 13.1 claims) have been reported and discussed earlier with probiotics and prebiotics as examples (1). Concerning especially gut health and probiotics and prebiotics, the European Food Safety Authority (EFSA), based on experiences gained with the evaluation of health claims, has published in 2011 a guidance document on scientific requirements for health claims related to gut and immune function to facilitate submission of application for the authorization of health claims (2). This guide addresses the beneficial effects and outcome measures that are acceptable for substantiation of claims in these areas (3).

The definition of probiotics has evolved over the years and the definition that is used most commonly is based on work of ILSI Europe and the WHO (4–11). The WHO expert group definition of probiotics states that probiotics are ‘live microorganisms which when administered in adequate amounts confer a health benefit on the host’ (7). The prebiotic definition according to FAO states that ‘A prebiotic is a non-viable food component that confers a health benefit on the host associated with modulation of the microbiota’ (7). The requirements for scientific substantiation of claims are also given in the EFSA Guidance document (3).

In the case of probiotics, the main health benefits appear to be associated with maintenance of healthy microbiota or improving the resilience of microbiota, for instance, by reducing the numbers or colonization of pathogenic bacteria or viruses and by maintaining and improving the intestinal integrity and barrier function. Specific probiotics may also be associated with other health benefits including desirable modulation of lactose intolerance, bowel function and gastrointestinal comfort, diarrhea prevention and symptom alleviation, and upregulation or downregulation of immune response. A probiotic claim or a prebiotic claim is any claim, which states, suggests, or implies that a probiotic food or a prebiotic food has particular characteristics relating to its origin, nutritional properties, and health (6–8).

The evidence required for a probiotic, a prebiotic, or a combination of a probiotic and a prebiotic for a health claims can be summarized as follows:

1. Characterization of the strain or each of the strains in a probiotic mix or combination or the prebiotic components;
2. Identification of the health relationship that is considered as a beneficial physiological effect to the target population (i.e. the general population or a defined part of it);
3. Demonstration of health effects in a normal healthy target population.

The demonstration of the health claims has to be established in human intervention studies, and the background needed is described in Fig. 1 (3–12).

**Fig. 1:**
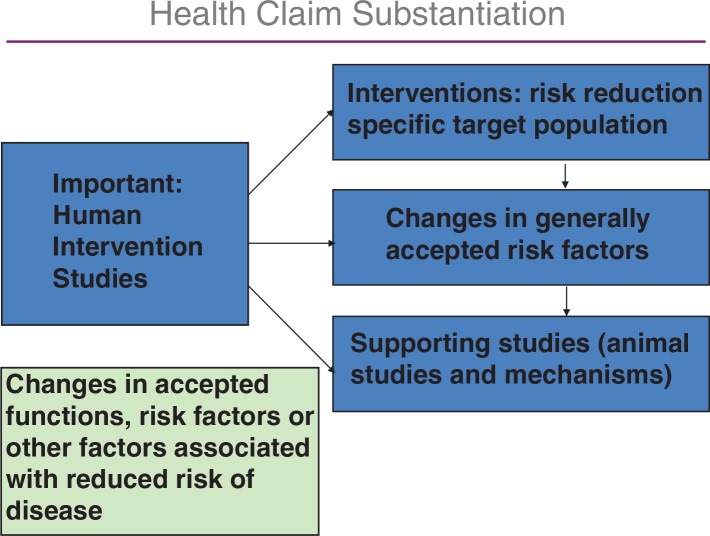
The components of health claim assessment.

A specific challenge for microorganisms and probiotics and prebiotics has been the definition of the health relationship. Often, the claimed health relationships have been too general to assess, or they were considered by EFSA to not be beneficial. For instance, many claims attempted to document that merely increasing the proportion of lactobacilli or bifidobacteria in the gut should be considered as a beneficial health effect. EFSA has not considered a change in bifidobacteria as generally agreed benefit, requiring further evidence associated with beneficial outcomes from human studies. So far, the evidence provided for substantiation of claims or available to the panel does not establish that increasing the numbers of any groups of microorganisms, including lactobacilli and/or bifidobacteria, is in itself a beneficial physiological effect. In fact, conceivably, the increase in bifidobacteria could even impact on potentially harmful strains; an example of this might be *Bifidobacterium dentium*, associated with dental caries (9).

Other examples of very general claims have been, for instance, statements such as ‘improves gut health’ or ‘boosts the immune system’, which should be more focused or identified for a specific target population.

Some claims have been judged as not eligible in accordance to the claims regulation as they pertained to treatment of pathological situations, rather than maintaining normal physiological conditions or reducing disease risk as demonstrated by the risk factors clearly indicated in the Regulation. Claims oriented at diseased people are outside the scope of the claims regulation. However, effects in specific health conditions can be accepted as evidence for effects in the general population. Some claims were constructed to reduce the risk of a disease, but the documents failed to identify risk factors. Several claims have failed because the research documents provided showed flaws in their design. Intervention studies were not always sufficiently randomized, measures for blinding subjects and/or observers were not always explained, and statistical analysis used was often either inappropriate or inadequate for a particular study design and for number of outcome measures. A flaw often encountered has been that many measures were studied, but statistical analysis failed to correct for multiple analysis. Another difficulty encountered by EFSA is that the evidence provided is insufficient to establish that the strain used in the studies is identical to the strain of the claim. There should always be sufficient information and definition of the strain used and the food constituents incorporated in the studies for substantiation of the claim.

Most probiotic studies in human subjects have been conducted in subjects who have been either ill or critically ill. A real challenge for scientists is posed by the requirement of the EU regulation where the health claims are clearly targeted for general healthy population or specific subgroups thereof, for example, elderly people, physically active subjects, or pregnant women. Such a requirement puts specific demands on human intervention studies. A review of such factors has been reported earlier (1).

Taken together, several challenges have appeared in the probiotic and prebiotic area and health claims have not been established according to the regulatory requirements. In spite of the extensive studies in the area, the focus has been outside the scope of the Regulation, as approved by the European Parliament. Thus, the research tools have to be redirected to areas that support future health claims. This can be achieved through focusing on the requirements of the regulation and through careful assessment of probiotic properties and related health outcomes. Also, the improved and more detailed guidance documents provided by EFSA will benefit all future research and applications. Learning from the experience of earlier assessment should make it possible to obtain health claims for probiotics and prebiotics in the future to provide the consumers reliable claims with new directions in helping them to make healthier choices.

## Conclusions and future developments

Based on the current regulation, the documentation for substantiation of health claims for probiotics and prebiotics is challenging but not impossible. Studies have to be designed using the guidance provided by the existing regulation and the guidance documents including specific opinions published by EFSA. It is likely that new developments will be directed to achieve the levels required and acceptable specific target population and the developments will enable new products with claims to be developed.
